# Crosstalk Between nNOS/NO and COX-2 Enhances Interferon-Gamma-Stimulated Melanoma Progression

**DOI:** 10.3390/cancers17030477

**Published:** 2025-01-31

**Authors:** Anika Patel, Shirley Tong, Moom R. Roosan, Basir Syed, Amardeep Awasthi, Richard B. Silverman, Sun Yang

**Affiliations:** 1 Biomedical and Pharmaceutical Sciences, Chapman University School of Pharmacy, Harry and Diane Rinker Health Science Campus, 9401 Jeronimo Road, Irvine, CA 92618, USA; 2 Department of Pharmacy Practice, Chapman University School of Pharmacy, Harry and Diane Rinker Health Science Campus, 9401 Jeronimo Road, Irvine, CA 92618, USA; 3 Department of Chemistry, Chemistry of Life Processes Institute, Northwestern University, Evanston, IL 60208, USA; 4 Department of Molecular Biosciences, Chemistry of Life Processes Institute, Northwestern University, Evanston, IL 60208, USA

**Keywords:** melanoma, interferon-gamma (IFN-γ), neuronal nitric oxide synthase (nNOS), nitric oxide (NO), cyclooxygenase-2 (COX-2), prostaglandin E2 (PGE_2_), nNOS inhibitors, celecoxib, programmed death-ligand 1 (PD-L1)

## Abstract

**Background/Objectives:** Interferon gamma (IFN-γ) in the melanoma tumor microenvironment plays opposing roles, orchestrating both pro-tumorigenic activity and anticancer immune responses. Our previous studies demonstrated the role of neuronal nitric oxide synthase (nNOS) in IFN-γ-stimulated melanoma progression. However, the underlying mechanism has not been well defined. This study determined whether the nNOS/NO and COX-2/PGE_2_ signaling pathways crosstalk and augment the pro-tumorigenic effects of IFN-γ in melanoma. **Methods**: Bioinformatic analysis of patient and cellular proteomic data was conducted to identify proteins of interest associated with IFN-γ treatment in melanoma. Changes in protein expression were determined using various analytical techniques including western blot, flow cytometry, and confocal microscopy. The levels of PGE_2_ and nitric oxide (NO) were analyzed by HPLC chromatography and flow cytometry. In vivo antitumor efficacy was determined utilizing a human melanoma xenograft mouse model. **Results**: Our omics analyses revealed that the induction of COX-2 was significantly predictive of IFN-γ treatment in melanoma cells. In the presence of IFN-γ, PGE_2_ further enhanced PD-L1 expression and amplified the induction of nNOS, which increased intracellular NO levels. Cotreatment with celecoxib effectively diminished these changes induced by PGE_2_. In addition, nNOS blockade using a selective small molecule inhibitor (HH044), efficiently inhibited IFN-γ-induced PGE_2_ and COX-2 expression levels in melanoma cells. STAT3 inhibitor napabucasin also inhibited COX-2 expression both in the presence and absence of IFN-γ. Furthermore, celecoxib was shown to enhance HH044 cytotoxicity in vitro and effectively inhibit human melanoma tumor growth in vivo. HH044 treatment also significantly reduced tumor PGE_2_ levels in vivo. **Conclusions**: Our study demonstrates the positive feedback loop linking nNOS-mediated NO signaling to the COX-2/PGE_2_ signaling axis in melanoma, which further potentiates the pro-tumorigenic activity of IFN-γ.

## Simple Summary

This study examines how two key signaling pathways in melanoma work together to promote tumor growth. Specifically, we focused on two enzymes—nNOS (neuronal nitric oxide synthase) and COX-2 (cyclooxygenase-2)—and their respective bioproducts, nitric oxide (NO) and prostaglandin E2 (PGE_2_). Our study revealed that interferon-gamma (IFN-γ), a cytokine that has been shown to stimulate melanoma progression, stimulates the expression of nNOS and COX-2, and subsequently results in the overproduction of NO and PGE_2_. In the presence of IFN-γ, the crosstalk between the COX-2/PGE_2_ and nNOS/NO pathways further augments the induction of PD-L1, an important protein associated with tumor immune suppression. Blocking COX-2 or nNOS using small molecule inhibitors effectively diminished the changes induced by IFN-γ, which could be a novel approach for melanoma therapy.

## 1. Introduction

Localized cutaneous melanoma has a 5-year survival rate of 100%, which drops dramatically to 35% upon metastasis [1]. Immune checkpoint inhibitors (ICIs) have shown strong clinical efficacy in reactivating T-cell-mediated antitumor immunity; however, the response rate to these novel immunotherapeutic treatments is limited to approximately 40–60% [2,3]. ICIs are also associated with severe immune-related adverse drug reactions, such as gastrointestinal toxicity, hepatitis, and endocrinopathies, that can impair patient compliance and often prevent completion of therapy [4,5]. Furthermore, the mutation rate of melanoma is one of the highest among different cancers, with mutations seen primarily in the BRAF, NRAS, and MEK genes, increasing the immunogenicity of tumor cells [3,6]. Thus, mutation-specific targeted therapies in melanoma often fail because cancer cells can rapidly develop resistance mechanisms, primarily by acquiring genetic mutations that bypass the targeted pathway, activate alternative signaling pathways, or modify the drug’s target, ultimately driving tumor progression [7,8]. Present challenges associated with melanoma pharmacotherapies highlight a need for developing innovative ways in which to treat this disease more effectively, including discovering new approaches to prevent progression and enhance the anticancer activity of immune checkpoint blockade.

Interferon-gamma (IFN-γ) is a critical cytokine in the activation of cellular immunity, enhancing the immune system’s ability to recognize and eliminate cancer cells [9]. However, its role in melanoma is more complex, as it can also contribute to tumorigenesis by increasing cancer cell fitness and fostering an immunosuppressive tumor microenvironment [10]. IFN-γ has been linked to melanoma disease progression, with clinical studies showing higher rates of relapse and mortality associated with its elevated presence [11]. While the precise mechanisms remain unclear, it is believed that IFN-γ stimulates the JAK/STAT signaling pathway in melanoma cells, leading to upregulation of immune checkpoint molecules like programmed death-ligand 1 (PD-L1), which helps melanoma cells evade immune attack [12]. It has also been hypothesized that IFN-γ induced PD-L1 expression may be dependent on NF-κB activity, which has been shown to be upregulated in human melanoma [13,14]. Additionally, chronic IFN-γ exposure can induce epigenetic alterations in melanoma cells, resulting in increased resistance to ICIs and further promoting tumor survival [3]. Thus, while IFN-γ is vital for immune responses, its prolonged or dysregulated activity in melanoma may enhance tumor progression and resistance to immunotherapy.

One of the well-studied risk factors for the development of melanoma is ultraviolet radiation (UVR) exposure. UVR has been shown to significantly increase nitric oxide (NO) levels in human skin, a process primarily driven by the enzymatic activity of nitric oxide synthases [15]. Excessive NO can cause DNA damage, promote carcinogenesis, and accelerate cell cycle progression [16]. Melanocytes, which originate from the neural crest, express the neuronal isoform of nitric oxide synthase (nNOS). Our previous studies demonstrated a crucial role of nNOS in regulating NO levels in melanoma cells, which is markedly induced by UVR and IFN-γ [15,17]. Overexpression of nNOS significantly correlates with melanoma progression [15,17]. Targeting nNOS with small molecule inhibitors has shown potent anti-melanoma effects both in vitro and in vivo, and effectively alleviated IFN-γ-induced PD-L1 expression in melanoma cells [17,18].

Cyclooxygenase-2 (COX-2) has also been implicated in the development and progression of melanoma. Elevated COX-2 correlates with PD-L1 expression in human malignant melanoma cell lines [2]. Studies also demonstrated that COX-2 was linked to the suppression of immune responses within the tumor microenvironment, including the inhibition of dendritic cells, natural killer cells, and T cells, thereby facilitating immune evasion [2]. COX-2 inhibition was shown to prevent human melanoma cell proliferation and induce cell death, independent of BRAF or NRAS mutations [2].

It has been well studied that NO stress upregulates COX-2 expression and directly stimulates COX-2 activity, leading to an increased production of PGE_2_ [19,20]. While a relationship between nitric oxide synthases (NOS) and COX-2 has been observed across various cancers, the specific synergistic mechanism in melanoma remains unclear. We hypothesize that there is crosstalk between nNOS/NO and COX-2/PGE_2_ signaling in the presence of IFN-γ in melanoma. Our study, for the first time, investigated the impact of the crosstalk on IFN-γ-stimulated PD-L1 expression.

## 2. Materials and Methods

### 2.1. Cell Lines, Reagents, and Antibodies

Human melanoma cell lines A375, SK-MEL-28, and WM115 from the American Type Culture Collection (ATCC; Manassas, VA, USA) were used in these studies. Cell lines were cultured in Dulbecco’s Modified Eagle’s Medium (DMEM; #30-2002; ATCC, Manassas, VA, USA) (A375) or Eagle’s Minimum Essential Medium (EMEM; #30-2003; ATCC, Manassas, VA, USA) (SK-MEL-28 and WM115) with 10% fetal bovine serum (FBS; #26140079; Gibco, Waltham, MA, USA).

The nNOS inhibitor used in these studies was HH044 (synthesized by Symphony Pharma Life Sciences and purified by Dr. Amardeep Awasthi in Richard Silverman’s lab, >98% purity), the specific COX-2 inhibitor used was celecoxib (169590-42-5; MedChemExpress, Monmouth Junction, NJ, USA), the NO donor was DetaNONOate (82120, Cayman Chemical Company, Ann Arbor, MI, USA), and the STAT3 inhibitor was napabucasin (#S7977; SelleckChem, Houston, TX, USA). IFN-γ was acquired from Gold Biotechnology (#1160-06-100, Olivette, MO, USA). The PGE_2_ and PGE_2_-d4 were obtained from Santa Cruz Biotechnology (#sc-201225C, Dallas, TX, USA) and Cayman Chemical Company (#314010, Ann Arbor, MI, USA), respectively.

### 2.2. Reverse Phase Protein Array and the Cancer Proteome Atlas Analysis

As previously described, whole cell lysates of human melanoma cells (A375, SK-MEL-28, and WM115) collected after treatment with control, IFN-α, and IFN-γ were subjected to Reverse Phase Protein Array (RPPA) analysis performed by the MD Anderson Functional Proteomics RPPA Core Facility [17]. The dataset was prepared, formatted, and analyzed using the *R statistical software 3.6.2*. To address cell line-specific variation, the data were adjusted using the *Combat* function from the “*sva*” R package [21]. The “*ASSIGN*” R package was used to identify the top differentially expressed proteins that can reliably predict IFN-γ treatment [22]. One of the top differentially expressed proteins, PD-L1, was further analyzed in an independent RPPA skin melanoma public dataset from The Cancer Proteome Atlas (TCPA) to evaluate the differential expression of PD-L1 in real melanoma patient samples [23].

### 2.3. Isolation of Protein and Western Blotting Analysis

Mouse monoclonal anti-human nNOS (#MAB2416; Novus Biologicals, Centennial, CO, USA), mouse monoclonal anti-human β-Actin (#8H10D10; Cell Signaling Technology, Danvers, MA, USA), rabbit monoclonal anti-human PD-L1 (#E1L3N; Cell Signaling Technology, Danvers, MA, USA), and rabbit monoclonal anti-human COX-2 (#D5H5; Cell Signaling Technology, Danvers, MA, USA) antibodies were used as primary antibodies, and horseradish peroxidase (HRP)-labeled anti-mouse and anti-rabbit (1:5000; 1:2000; Cell Signaling Technology, Danvers, MA, USA) were utilized as secondary antibodies for western blotting analysis. Protein isolation and the western blotting protocol have been described previously [17]. The results were visualized using the Bio-Rad ChemiDoc XRS+ System (Bio-Rad, Hercules, CA, USA).

### 2.4. Detection of PD-L1 Expression Levels Using Flow Cytometry and Confocal Microscopy

The PD-L1 antibodies used were conjugated with Alexa Fluor 647 and 488 (#41726S; #24048S; Cell Signaling Technology, Danvers, MA). Cells were collected after treatment and fixed with 4% paraformaldehyde in 1× PBS for 10 min on ice, followed by incubation in a PD-L1 antibody dilution (0.5% BSA in 1× Hank’s Balanced Salt Solution (HBSS)) for 2 h at room temperature before flow cytometry analysis. Mean fluorescence was ascertained using the BD FACSymphony A1 Cell Analyzer (BD Biosciences, Franklin Lakes, NJ, USA).

For confocal microscopy imaging, cells were prepared following the immunofluorescence protocol outlined previously and visualized on the Nikon Eclipse Ti2-E confocal microscopy system (Nikon, Melville, NY, USA) [17].

### 2.5. Detection of Intracellular Nitric Oxide Levels by Flow Cytometry

Human melanoma cells were collected after treatment and incubated in 1× HBSS containing DAF-2 diacetate (#85165; Cayman Chemical Company, Ann Arbor, MI, USA) for 30 min, followed by a 30-min incubation in serum-free media at 37 °C. Cells were resuspended in 1× HBSS and analyzed via flow cytometry.

### 2.6. Detection of PGE_2_ Level by LC-MS/MS

Human melanoma cells were seeded and incubated in various treatments in DMEM media without serum for 48 h. The supernatant was collected for PGE_2_ level analysis. The prostaglandin extraction and LC-MS/MS methods were adapted from the protocol by Cao et al. [24]. The culture medium was collected, and 20 μL PGE_2_-d4 (100 ng/mL each) was added as the internal standard. For extraction, 40 μL 1M citric acid and 2 mL hexane/ethyl acetate (1:1, *v*/*v*) were added, and the sample was vortexed for 1 min. After centrifuging at 2500× *g* for 5 min, the upper organic phase was collected, evaporated, and reconstituted in 200 μL methanol/10 mM ammonium acetate buffer, pH 8.5 (1:3 *v*/*v*), for LC-MS/MS analysis using the Bruker EVOQ LC-TQ (Bruker; Billerica, MA, USA).

The prostaglandins were separated on the Waters Xterra Phenyl analytical column (2.1 × 50 mm, 5 μm particle size) using mobile phases of 0.1% formic acid in water (A) and 100% methanol (B). Electrospray ionization (ESI) in negative mode was used for multiple reaction monitoring (MRM) and quantification of analytes at 4800 V spray voltage. The cone temperature and heated probe temperature were set to 350 °C. The precursors to the product ion transition were as follows: *m*/*z* 351 → 271, 315, 333 for PGE_2_ and *m*/*z* 355 → 275, 319, 337 for PGE_2_-d4. The LC gradient was 20% B from 0–0.3 min, 20% to 50% B from 0.3–2.0 min, 50% B from 2.0–2.1 min, 50% to 20% B from 2.1–2.2 min, and 20% B from 2.2–3.5 min.

### 2.7. MTT Colorimetric Assay

Cell viability was determined as described previously [25]. IC_50_ was analyzed using GraphPad Prism 9 through log(inhibitor) versus normalized response analysis.

### 2.8. Detection of PGE_2_ Levels in Tumors Using a Syngeneic Murine Melanoma Mouse Model

The animal studies were conducted with approval from the Institutional Animal Care and Use Committee (IACUC) of Chapman University. Male DBA/2 mice were purchased from Charles River (Wilmington, MA, USA) and housed in the Chapman University vivarium. To induce melanoma tumor growth, the mice were injected subcutaneously with Cloudman S91 murine melanoma cells (1 × 10^5^ in 100 μL 50% Matrigel/F12K medium) (CB354248, Corning, Corning, NY, USA). The mice were randomized into the following treatment groups: control (normal saline 0.1 mL/10 g i.p.), HH044 10 mg/kg/day i.p., and celecoxib (a selective COX-2 inhibitor) 50 mg/kg/day p.o. and treated for 24 days. Mice were euthanized when tumor volume reached 2000 mm^3^ calculated as [Length × (Width^2^)]/2. An immunocompetent mouse model was used for PGE_2_ analysis as it provides intact immune responses with tumor-infiltrating lymphocytes (TILs), which may mimic the tumor microenvironment more closely as seen in patients.

At the end of the study, tumors were collected from the mice for PGE_2_ level analysis. PGE_2_ was extracted by homogenizing 100 mg of tumor tissue in 500 μL of Milli-Q water. The homogenate was mixed with 1 mL hexane/ethyl acetate (1:1, *v*/*v*) and vortexed. After centrifugation at 2500× *g* for 5 min, the supernatant was collected, and PGE_2_ was extracted as outlined above in Section 2.6.

### 2.9. Evaluation of In Vivo Antitumor Activities Using a Human Melanoma Xenograft Mouse Model

With the approval of the Chapman University IACUC, nude mice (*Nu/Nu*, female) purchased from Charles River (Wilmington, MA, USA) were inoculated subcutaneously with human melanoma A375 cells (1 × 10^5^ in 100 μL 50% Matrigel/DMEM media). Only female mice were utilized for this study, as A375 cells were originally isolated from a female malignant melanoma patient. After 24 h, the mice were randomized into different groups. The control group received 0.5% carboxymethylcellulose (0.1 mL/10 g, *n* = 7), and the treatment group was administered celecoxib (*n* = 7) at a dose of 50 mg/kg in 0.5% carboxymethylcellulose via oral gavage daily. Tumor volume (mm^3^) was measured twice weekly and calculated as [Length × (Width^2^)]/2. Once the tumor size reached 2000 mm^3^, the mice were euthanized, marking the endpoint of the survival study.

### 2.10. Statistical Analyses

Each experiment was conducted at least three times, and the data presented represent the means ± SD from a minimum of three independent experiments. Statistical analysis was conducted using the Student’s *t*-test or one-way ANOVA with pairwise multiple comparisons. A *p*-value of less than 0.05 was considered statistically significant. Kaplan-Meier survival analysis was utilized to determine cumulative survival probability for the in vivo study.

## 3. Results

### 3.1. PD-L1 and COX-2 Identified as Differentially Expressed Proteins Predictive of IFN-γ Treatment in Melanoma

**Figure 1:**
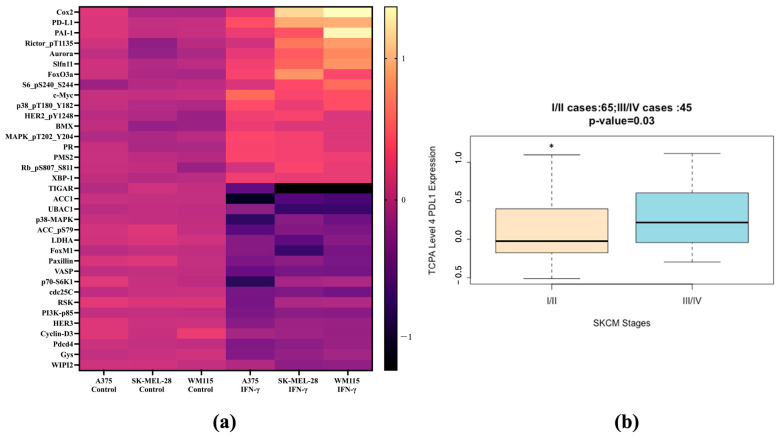
Differentially expressed proteins that can predict IFN-γ treatment in melanoma. (**a**) Reverse Phase Protein Array data were used to identify proteins with significant changes in expression after IFN-γ (250 units/mL) treatment compared to untreated cells across three human melanoma cell lines. The color scale indicates log2 median-centered intensity in protein expression for each cell line. The top 35 genes used to predict IFN-γ treatment are shown. (**b**) The Cancer Proteome Atlas Level 4 PD-L1 protein expression data for a total of 110 patients who had skin cutaneous melanoma (SKCM) were analyzed to compare patients with advanced disease (stage III/IV, *n* = 45) to stage I/II melanoma patients (*n* = 65, *, *p* = 0.03).

The top 35 differentially expressed proteins that accurately predicted IFN-γ treatment in human melanoma cells are presented in Figure 1a as log2 median-centered intensity in protein expression. The top two ranked proteins indicative of the differential effects between IFN-γ and IFN-α were COX-2 and PD-L1 (Supplementary Table S1). Within this dataset, these protein expression signatures accurately predicted IFN-γ activity over that of IFN-α across the three melanoma cell lines (Supplementary Table S2). The role of PD-L1 was further investigated in patients with various stages of cutaneous melanoma. TCPA protein expression data for 110 patients showed PD-L1 expression was significantly higher in patients with advanced melanoma (Stage III/IV) compared to patients with an early stage of the disease (Stage I/II) (Figure 1b).

### 3.2. Induction of PD-L1 Expression by IFN-γ Was Increased by PGE_2_ and Inhibited by Celecoxib

**Figure 2:**
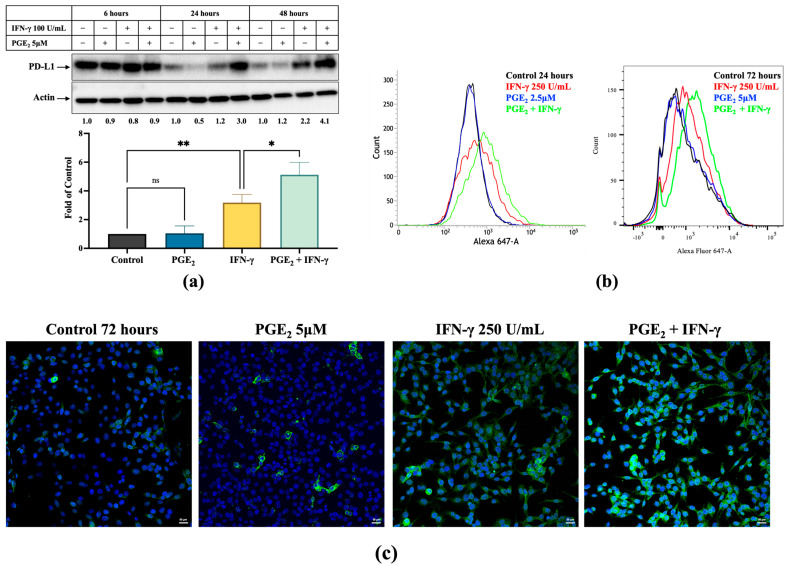

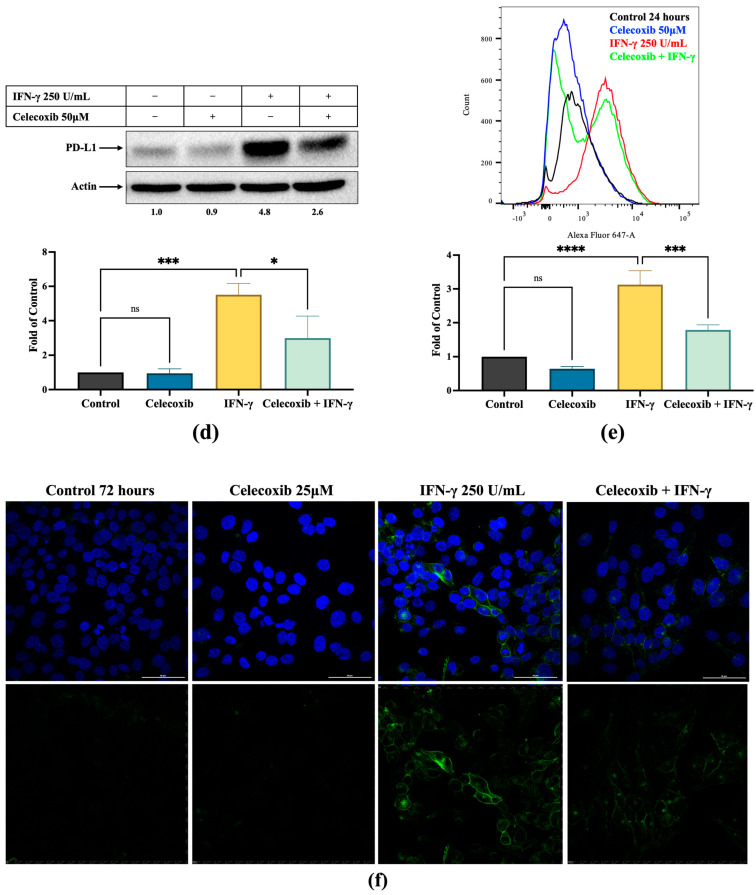
Effects of PGE_2_ and COX-2 inhibition on PD-L1 expression in human melanoma. (**a**) Immunoblots detected the effects of PGE_2_ on PD-L1 expression in human melanoma A375 cells in the presence and absence of IFN-γ. β-actin was utilized to normalize loaded protein. The bar graph represents the 24 h results (*n* = 3). Full-length blots of manuscript is shown in Figure S3. (**b**,**c**) Representative flow cytometry and confocal microscopy images of A375 cells treated with PGE_2_ with and without IFN-γ for 24 and 72 h, respectively. Cells were stained for expression of PD-L1 (Alexa Fluor 488, green) and nuclei (DAPI, blue). Confocal imaging was taken at 20× magnification; scale bar: 50 µm. (**d**) Celecoxib cotreatment reduced PD-L1 levels compared to IFN-γ treatment alone. Full-length blots of manuscript is shown in Figure S4. (**e**) Representative flow cytometry images of PD-L1 mean fluorescence and (**f**) images of immunofluorescent staining of PD-L1 by confocal microscopy (60× magnification, scale bar: 50 µm) after celecoxib treatment with and without IFN-γ. Cells were stained for expression of PD-L1 (Alexa Fluor 488, green) and nuclei (DAPI, blue). * *p* < 0.05, ** *p* < 0.01, *** *p* < 0.001, **** *p* < 0.0001, ns—not significant.

As shown in Figure 2a, PD-L1 expression was significantly induced with PGE_2_ (5 μM) cotreatment compared to IFN-γ treatment alone (250 units/mL) (*p* < 0.05). PD-L1 levels increased to 3.0-fold and 4.1-fold of control with cotreatment at 24 h and 48 h, respectively, while PD-L1 levels with IFN-γ treatment alone were only 1.2- and 2.2-fold of control. The basal level of PD-L1 expression at 24 and 48 h was reduced compared to the 6-h timepoint in serum-free culture medium. However, cotreatment of IFN-γ and PGE_2_ significantly increased PD-L1 expression compared to control and sustained PD-L1 expression at high levels over 48 h. Similar inductions were also observed with flow cytometry (Figure 2b) and confocal microscopy (Figure 2c). Marked increases in fluorescence were evident after PGE_2_ and IFN-γ cotreatment compared to IFN-γ alone at 24 h (2.8- and 1.7-fold of control, respectively) and 72 h (1.5- and 1.2-fold of control, respectively) (Figure 2b). However, PGE_2_ treatment itself did not have a marked effect on PD-L1 expression in comparison to control in any of the above experiments.

After celecoxib (50 μM) treatment for 48 h, a significant decrease in IFN-γ-mediated PD-L1 expression was observed by immunoblot (Figure 2d). In comparison to IFN-γ alone, cotreatment with celecoxib decreased PD-L1 expression from 4.8-fold to 2.6-fold of control. Flow cytometry analysis also showed diminished PD-L1 expression with celecoxib coincubation (Figure 2e, *p* < 0.001), consistent with confocal microscopy imaging shown in Figure 2f.

### 3.3. PGE_2_ Induces nNOS Expression in Melanoma Cells, Which Is Inhibited by Celecoxib

**Figure 3:**
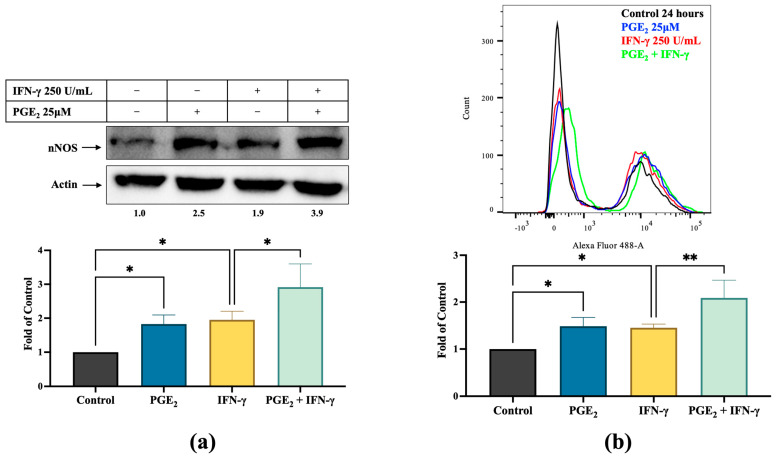

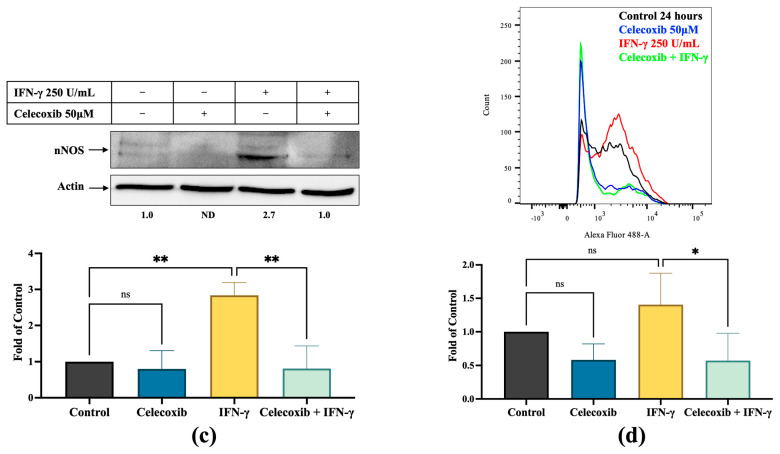
Effects of PGE_2_ and COX-2 inhibition on nNOS expression and NO levels in human melanoma cells. (**a**) A375 cells were treated with PGE_2_ (25 μM) with and without IFN-γ (250 units/mL) for 24 h, followed by immunoblot analysis of nNOS expression. β-actin was used to normalize loaded protein. Full-length blots of manuscript is shown in Figure S5. (**b**) Representative flow cytometry images of intracellular NO levels increased by PGE_2_ in the presence and absence of IFN-γ as detected using a DAF fluorescence probe. (**c**) Immunoblot analysis of nNOS expression levels after celecoxib treatment (50 μM) for 48 h. Full-length blots of manuscript is shown in Figure S6. (**d**) Celecoxib cotreatment reduced intracellular NO levels in the presence of IFN-γ in A375 cells. The impact of different treatments on NO levels in human melanoma SK-MEL-28 cells is shown in Supplementary Figure S10. * *p* < 0.05, ** *p* < 0.01, ns—not significant.

To determine whether nNOS-mediated NO signaling crosstalks with the COX-2-PGE_2_ signaling axis, we incubated A375 cells with PGE_2_ for 24 h in the presence and absence of IFN-γ and determined the changes in nNOS expression and intracellular NO levels. nNOS expression (Figure 3a) and NO production (Figure 3b) were significantly induced by PGE_2_ incubation compared to control (2.5-fold and 1.5-fold, respectively, *p* < 0.05). In the presence of IFN-γ, PGE_2_ further increased nNOS expression to 3.9-fold of control, and intracellular NO levels were elevated from 1.5-fold to 2.1-fold (*p* < 0.005).

Such inductions were markedly diminished by celecoxib cotreatment. The relative nNOS expression levels were decreased from 2.7-fold of control to approximately baseline by celecoxib cotreatment in the presence of IFN-γ (Figure 3c, *p* < 0.01 compared to IFN-γ alone). Consistently, IFN-γ-stimulated intracellular NO production was also reduced by celecoxib to levels even below the baseline (57% of control, Figure 3d).

### 3.4. NO Increases COX-2 Expression and PGE_2_ Levels in Melanoma Cells, Which Is Inhibited by nNOS Inhibitor HH044

**Figure 4:**
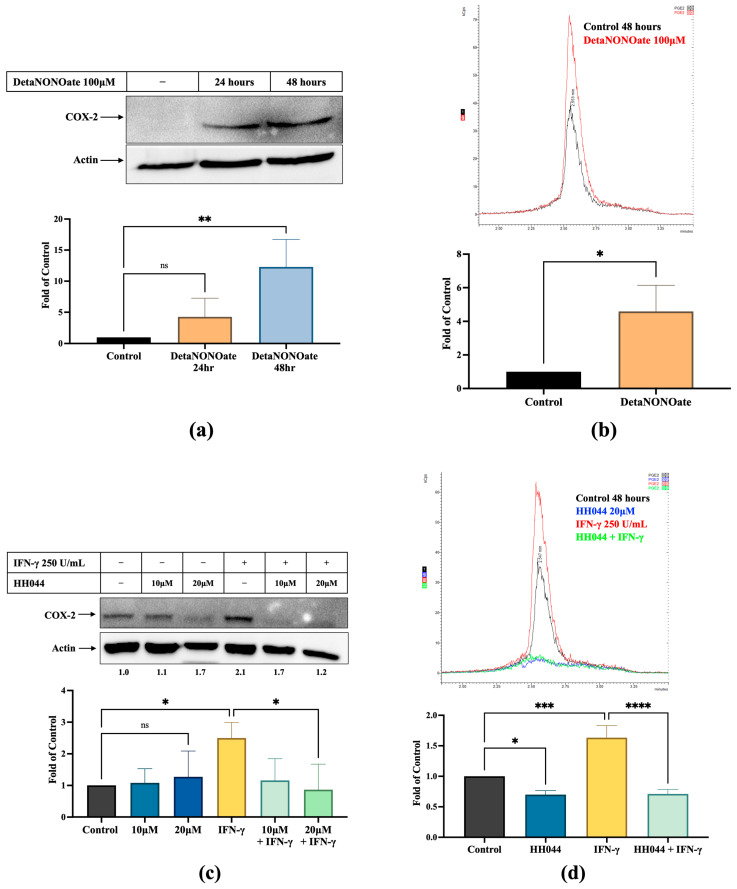
Effects of NO stress on COX-2 expression and PGE_2_ levels in human melanoma. NO stress significantly induced COX-2 expression (**a**) and PGE_2_ production (**b**) in melanoma cells. Full-length blots of manuscript is shown in Figures S7 and S8. A375 cells were cultured in serum-free DMEM medium with DetaNONOate 100 μM for 24 and 48 h. After 48 h, the media was collected for analysis of PGE_2_ levels using LC-MS/MS. The internal standard PGE_2_-d4 chromatogram is shown in Supplementary Figure S1. Cotreatment with nNOS inhibitor HH044 effectively diminished the induction of COX-2 (**c**) and PGE_2_ production (**d**) by IFN-γ in SK-MEL-28 and A375 cells, respectively. DMEM media was collected after treatment with HH044 20 μM and IFN-γ 250 units/mL for 48 h. The corresponding PGE_2_-d4 chromatogram is shown in Supplementary Figure S2. The impact of different treatments on PGE_2_ levels in human melanoma SK-MEL-28 cells is shown in Supplementary Figure S11. * *p* < 0.05, ** *p* < 0.01, *** *p* < 0.001, **** *p* < 0.0001, ns—not significant.

A375 cells were treated with a NO donor, DetaNONOate, for 24 and 48 h before collecting whole cell lysates for immunoblot analysis of COX-2 expression levels. DetaNONOate prominently increased COX-2 expression at 24 h and 48 h (Figure 4a), indicating a strong induction of COX-2 by NO stress in a possible time-dependent manner. Consistent with COX-2 induction, a marked increase in PGE_2_ was detected in culture media after incubation with DetaNONOate for 48 h (Figure 4b).

As shown in Figure 4c, HH044 cotreatment significantly truncated the induction of COX-2 by IFN-γ (2.1-fold of control to 1.2-fold of control at the 20 μM dose). However, we did not observe any significant changes in COX-2 expression by HH044 alone. Exposure to IFN-γ also significantly increased PGE_2_ production to 1.6-fold of control. nNOS blockade using HH044 effectively diminished the elevation of PGE_2_ in the presence of IFN-γ to below the basal level (71% of control) (Figure 4d). HH044 treatment alone also reduced the PGE_2_ level to below control (70% ± 6.6%, *p* < 0.05).

### 3.5. STAT3 Inhibitor Napabucasin Decreases IFN-γ-Stimulated COX-2 Expression

**Figure 5:**
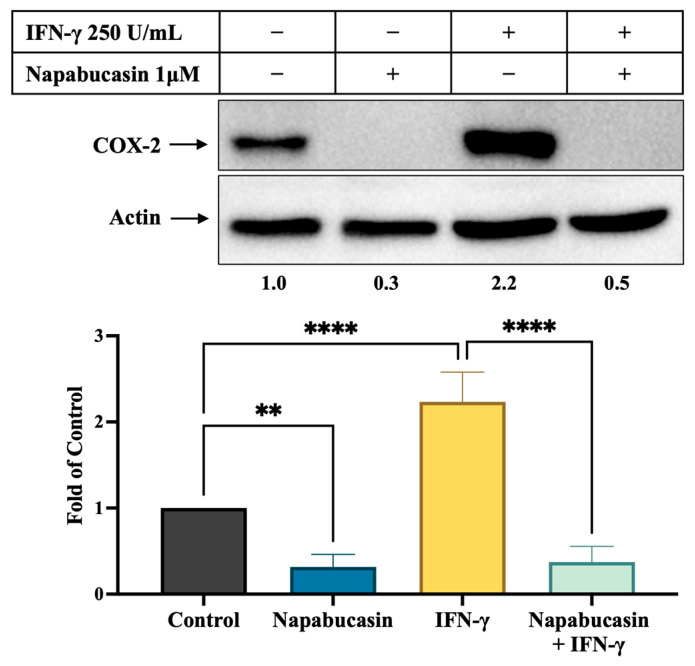
Effects of STAT3 inhibitor napabucasin on COX-2 expression in melanoma cells. SK-MEL-28 cells were treated with napabucasin 1 μM with and without IFN-γ 250 units/mL for 72 h and analyzed using immunoblot. Expression levels of COX-2 were normalized via β-actin. ** *p* < 0.01, **** *p* < 0.0001. Full-length blots of manuscript is shown in Figure S9.

SK-MEL-28 cells were treated with 1 μM napabucasin alone and in combination with IFN-γ 250 units/mL. Napabucasin alone significantly decreased COX-2 expression to 30% of the control level (*p* < 0.005, Figure 5). In the presence of IFN-γ, napabucasin effectively reduced COX-2 levels from 2.2-fold of control to 50% of control (*p* < 0.0001 compared to IFN-γ alone).

### 3.6. Celecoxib Increases the Cytotoxicity of nNOS Inhibition in Human Melanoma Cells

**Figure 6:**
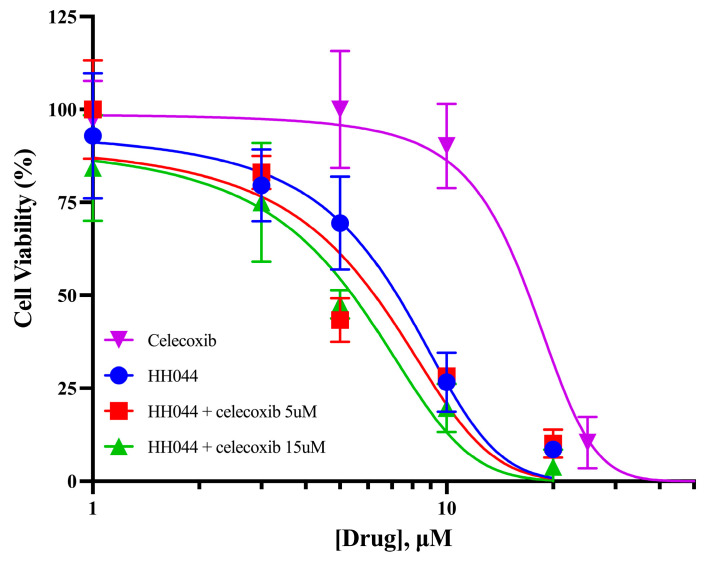
Celecoxib enhances the cytotoxicity of nNOS inhibitor HH044 in melanoma cells. IC_50_ was determined using GraphPad Prism, detected by MTT colorimetric analysis. A375 cells were treated with various concentrations of HH044 and celecoxib for 72 h, and viable cells were measured by absorbance at 595 nm. All IC_50_ values are included in Supplementary Table S3.

We further determined the cytotoxicity of HH044 and celecoxib in human melanoma cells alone and in combination. The IC_50_ values of HH044 and celecoxib were 6.58 µM and 16.7 μM, respectively, in A375 melanoma cells (Figure 6). In the presence of celecoxib 5 µM (30% of the IC_50_), the IC_50_ of HH044 reduced to 5.38 µM (Figure 6, Supplementary Table S3).

### 3.7. In Vivo Tumor PGE_2_ Levels Using an Immunocompetent Syngeneic Melanoma Mouse Model

**Figure 7:**
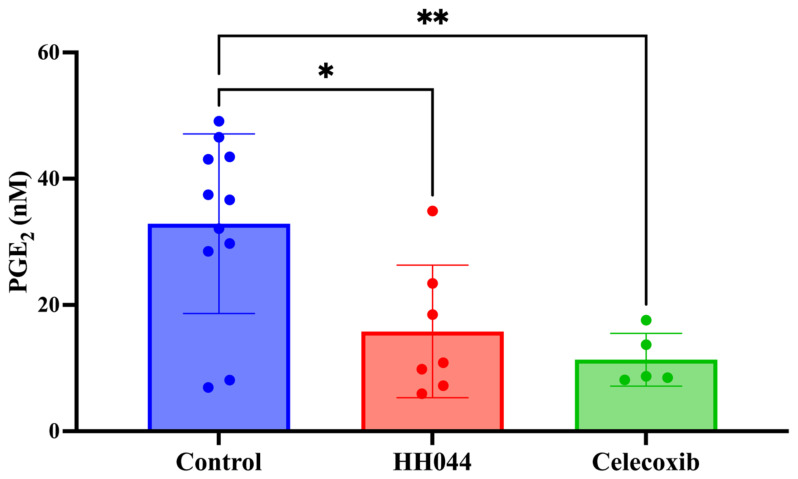
The nNOS inhibitor HH044 decreased tumor PGE_2_ levels in vivo. DBA/2 male mice were injected with Cloudman S91 cells to induce tumor growth. Mice were then randomized into different groups (vehicle control, HH044 10 mg/kg i.p., and celecoxib 50 mg/kg p.o. daily for 24 days). At the end of the study, tumors were collected and processed for PGE_2_ analysis using LC/MS-MS. * *p* < 0.05, ** *p* < 0.01, compared to control.

DBA/2 mice were inoculated with Cloudman S91 cells to induce tumor growth and were treated with control, HH044, and celecoxib daily for 24 days. After completion of treatment, tumors were collected and analyzed for PGE_2_ levels. Fewer mice in the treatment groups reached the euthanization criteria, and some tumors were too small for analysis at the end of the study. As shown in Figure 7, the control tumors had a mean PGE_2_ level of 32.88 nM. PGE_2_ in the tumors treated with HH044 and celecoxib decreased to 48% and 34% of the control (15.80 nM and 11.33 nM, respectively) (Figure 7).

### 3.8. In Vivo Anti-Melanoma Activity of Celecoxib Using a Xenograft Mouse Model

**Figure 8:**
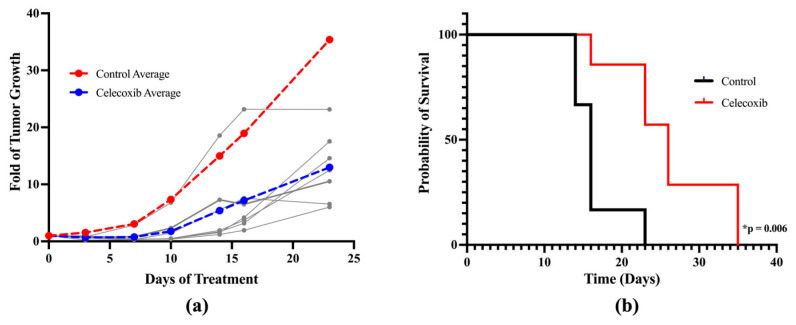
Celecoxib showed potent anti-melanoma activity in vivo. Nude mice were injected with human melanoma A375 cells to induce tumor growth and were treated with celecoxib 50 mg/kg/day p.o. for 23 days. Tumors were measured biweekly. (**a**) Tumor growth is presented as the fold of tumor volume on Day 0 (5 days post inoculation, and the day before starting treatment) for each individual mouse. The average fold of tumor growth is shown in red for the control (*n* = 7) and in blue for celecoxib (*n* = 7). The tumor growth curves of individual mice treated with celecoxib are represented in gray. The tumor growth curve in volume is presented in Supplementary Figure S12. (**b**) Kaplan–Meier curve for overall survival in nude mice treated with control and celecoxib. * *p* < 0.05 compared to control.

Nude mice were inoculated with human melanoma A375 cells to induce tumor growth and received either vehicle control (0.5% carboxymethylcellulose) or celecoxib treatment for 23 days (50 mg/kg/day p.o.). As shown in Figure 8a, celecoxib effectively inhibited tumor growth compared to the control, with no significant systemic toxicities noted. On day 23, the average tumor volume was reduced to 37% of the control with celecoxib treatment. No significant changes in body weight were observed (22.5 g in the control and 23.4 g in the celecoxib group on average). Further survival analysis demonstrated that celecoxib extended the median survival to 26 days compared to 16 days in the control group (Figure 8b, *p* < 0.05).

## 4. Discussion

**Figure 9:**
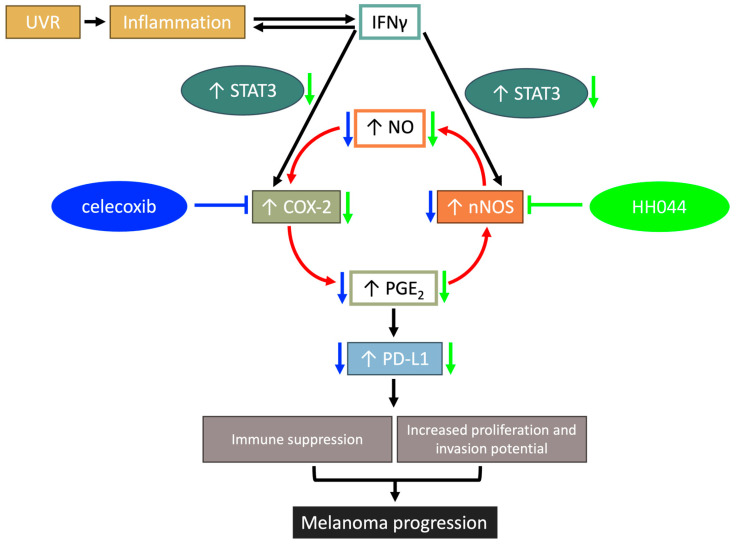
A schematic representation of the crosstalk between the nNOS/NO and COX-2/PGE_2_ signaling pathways, which enhanced IFN-γ-induced PD-L1 expression in melanoma. IFN-γ has been implicated as a pro-tumorigenic cytokine attributed to melanoma progression. Our study has demonstrated that the nNOS/NO and COX-2/PGE_2_ pathways are activated in the presence of IFN-γ, both of which generate proinflammatory molecules. The enzymatic product of COX-2 activity, PGE_2_, induced the expression of nNOS in melanoma cells, while NO produced by nNOS further increased the expression of COX-2. As a result, this feedforward loop amplifies the pro-tumorigenic effects of IFN-γ in melanoma by inducing PD-L1, leading to immune suppression within the tumor microenvironment. Blockade of COX-2 and/or nNOS using selective inhibitors may be a promising approach for melanoma therapy, which effectively alleviates the pro-tumorigenic effects of IFN-γ in melanoma cells without inhibiting the essential immune cell intrinsic function of IFN-γ in tumor immunosurveillance. The blue downward arrows indicate the effect of COX-2 inhibitor celecoxib and green downward arrows indicate the effect of the nNOS inhibitor HH044.

Our study demonstrated the intricate crosstalk between the COX-2/PGE_2_ and nNOS/NO signaling pathways and their contribution to IFN-γ-stimulated melanoma progression. IFN-γ has exhibited pro-tumorigenic effects in its upregulation of both COX-2, shown in this study, as well as nNOS, shown in our previous studies [17]. Activation of COX-2 and nNOS is associated with enhanced PD-L1 expression in the presence of IFN-γ, which contributes to evasion of immune surveillance. Our data suggests a crosstalk between the COX-2/PGE_2_ and nNOS/NO pathways, creating a feedforward loop implicated in amplifying the effects of IFN-γ in melanoma. The results, along with accumulating evidence [10,12,17,26,27], provide insight into the pro-tumorigenic activity of IFN-γ and underscore the potential of targeting nNOS and COX-2 as promising pharmacotherapeutic approaches for melanoma by inhibiting IFN-γ-stimulated PD-L1 expression (Figure 9).

### 4.1. COX-2, PGE_2_, and Melanoma

COX-2 has been implicated as a potential biomarker and therapeutic target in the treatment of melanoma. COX-2 enzymatic activity plays a central role in chronic inflammation, which may lead to the activation of various tumor-promoting signaling pathways disseminating both tumor development and progression [28,29,30]. It has been shown to be expressed in many tumor types, correlating with invasiveness and prognosis, while high levels of COX-2 have been detected in various human and murine malignant melanoma cell lines [31,32,33,34,35,36]. Ptgs1-negative Ptgs2-negative Braf^V600E^-negative tumors were also shown to be noticeably smaller than COX-2-sufficient tumors [37].

Our bioinformatics analysis further highlighted induction of COX-2 as being a strong predictor of IFN-γ treatment in melanoma. PGE_2_ produced as a consequence of COX-2 enzymatic activity is a key mediator of chronic inflammation. Within the tumor microenvironment, inflammation can have a detrimental effect promoting tumor development and progression, metastatic dissemination, and treatment resistance [38,39]. IFN-γ, on the other hand, is produced by activated T cells to elicit an antitumor response, but in turn can activate certain immune cells that produce other pro-inflammatory cytokines [27]. Furthermore, high levels of PGE_2_ within the tumor microenvironment may also lead to tumor immunosuppression through the regulation of certain T cell subtypes [40]. As such, a complex interplay between inflammation and immunosuppression may in turn lead to melanoma progression. Our study showed a strong induction of COX-2 and increased production of PGE_2_ in the presence of IFN-γ. These data suggest that IFN-γ-induced melanoma progression may be instigated in relation to COX-2 expression and activity.

The COX-2 selective inhibitor celecoxib is currently being studied in a phase II trial as a possible therapeutic strategy for the treatment of HLA-A2+ refractory melanoma in combination with autologous alpha-type-1 polarized dendritic cells (alphaDC1)/TBVA cell-based treatment, rintatolimod, and recombinant interferon alpha-2 [41]. Our study correspondingly showed potent anticancer activity of celecoxib both in vitro and in vivo by reducing PGE_2_ production.

### 4.2. Crosstalk Between nNOS and COX-2 Plays an Important Role in IFN-γ-Stimulated Melanoma Progression

UVR is a well-studied risk factor of melanomagenesis, which is also a known causative stimulus of NO production in human skin [15,42]. In melanocytes, NO stimulates melanocyte proliferation and survival, promotes oxidative stress which can damage DNA and alter cellular signaling pathways, and modulates the tumor microenvironment by disseminating angiogenesis and suppressing immune responses [16,43]. Our previous studies demonstrated that nNOS is highly abundant in melanoma compared to melanocytes and that expression increases with disease stage in melanoma biopsies, highlighting the critical role of nNOS-mediated NO signaling in melanoma [15]. Exposure to IFN-γ and UVR is shown to induce nNOS expression and increase NO production [17]. Likewise, repeated UVA and UVB skin damage has also been shown to trigger the production of arachidonic acid in human keratinocytes furthering DNA damage and COX-2-mediated PGE_2_ production [30]. An in vivo study showed that COX-2-deficient mice and those treated with COX-2 inhibitors had significantly lower risk of developing UV-induced skin carcinogenesis [44].

Both NO production and COX-2 expression levels have been identified as poor prognostic indicators for survival in many aggressive cancers [19]. In estrogen receptor-negative breast cancer, dual inhibition of inducible nitric oxide synthase (iNOS) and COX-2 significantly reduced tumor volume in xenograft murine models, suggesting possible benefit from dual targeting of these enzymes [45]. Furthermore, a study done on breast cancer patients identified that tumors expressing high levels of both iNOS and COX-2 had dismal survival outcomes compared with over 95% survival in patients with low expression of both enzymes [45]. However, the mechanistic basis of the synergistic effects of NO and COX-2 in melanoma has yet to be determined.

Bioinformatic analyses revealed that PD-L1 expression was a top predictor of IFN-γ treatment, highlighting the importance of this protein in the subsequent dissemination of IFN-γ-induced melanoma progression. Our selective nNOS inhibitors have an exciting antitumor immunoregulatory mechanism through the significant reduction of IFN-γ-induced PD-L1 expression [17]. In conjunction, COX-2 expression has been shown to correlate with and modulate PD-L1 expression in melanoma cells in vitro, and celecoxib decreased PD-L1 expression significantly [2]. While IFN-γ itself has been shown to upregulate PD-L1 levels in melanoma [17], our study has highlighted a stronger induction of PD-L1 after cotreatment with PGE_2_ or DetaNONOate compared to IFN-γ alone. This analysis demonstrated that COX-2/PGE_2_ and nNOS/NO signaling, activated by IFN-γ, interact in a feedforward manner, ultimately amplifying the induction of PD-L1 in melanoma cells. The crosstalk between these two signaling pathways contributes to IFN-γ-stimulated disease progression by facilitating the development of an immunosuppressive microenvironment. Blocking the enzymatic activity of nNOS and COX-2 using selective inhibitors effectively diminished IFN-γ-induced PD-L1 expression and exhibited promising in vivo anti-melanoma activity.

Immunotherapy using immune checkpoint inhibitors has now become first-line treatment for advanced melanoma [46]. COX-2 and PD-1 blockade may improve the anti-melanoma activities of ICIs by both inhibiting PD-L1 expression and subsequent binding to T-cell PD-1. This has been supported by an earlier study, which showed complementary antitumor activity when combining COX-2 inhibitors with anti-PD-1 in a Braf^V600E^ melanoma mouse model [37]. An earlier clinical study showed that PD-L1 expression was associated with better prognosis and response to immunotherapy in melanoma patients [47]. However, patients with PD-L1 < 1% still benefit from a combination of anti-PD-1 and anti-CTLA-4 therapy with improved progression-free survival [48]. In our future studies, we will use an immune-competent syngeneic mouse melanoma model to determine if cotreatment with nNOS inhibitors improves the antitumor activities of ICIs.

### 4.3. The Role of STAT3 Induced by IFN-γ in the Tumor Microenvironment

IFN-γ is known to activate STAT3, a nuclear transcription factor implicated in playing an essential role in the development and progression of melanoma [49,50]. STAT3 is constantly activated in melanoma cells, and highly metastatic melanomas are shown to have higher levels of activated STAT3 [51,52]. In BRAF^V600E^-positive human melanoma, activation of the JAK2/STAT3 pathway led to increased COX-2 expression, sustaining chronic inflammation and promoting tumor evasion [53,54]. The JAK/STAT pathway has also been proposed as a potential mechanism of melanoma PD-L1 expression regulated by IFN-γ production in the tumor microenvironment [12]. Our study showed that inhibition of STAT3 proficiently reduced COX-2 expression both alone and induced by IFN-γ in melanoma, consistent with previous reports of STAT3 occupying and regulating expression at the COX-2 promoter [55]. We previously reported that nNOS inhibition effectively reduced IFN-γ-induced STAT3 expression [17], which may, at least partially, explain how HH044 cotreatment diminished IFN-γ-induced COX-2 and subsequent PGE_2_ production. Further studies are warranted to determine how nNOS-mediated NO regulates the IFN-γ/STAT3 signaling axis in melanoma cells within the tumor microenvironment.

## 5. Conclusions

Our study demonstrated that crosstalk between the nNOS/NO and COX-2/PGE_2_ pathways amplifies IFN-γ-stimulated PD-L1 expression in melanoma cells, potentially contributing to the development of an immunosuppressive microenvironment. Targeting COX-2 and nNOS provides a novel therapeutic approach for melanoma therapy by inhibiting the pro-tumorigenic activity of IFN-γ.

## Data Availability

The original contributions presented in this study are included in the article/Supplementary Material. Further inquiries can be directed to the corresponding author.

## Supplementary Materials

The following supporting information can be downloaded at https://www.mdpi.com/article/10.3390/cancers17030477/s1, Table S1: Top 35 protein probes and their coefficients from the *ASSIGN* prediction; Table S2: Predicted interferon-gamma treatment activity in three cell lines treated with vehicle, IFN-α, and IFN-γ; Table S3: IC50 values of HH044 and celecoxib from MTT assay in A375 human melanoma cells; Figure S1: Internal standard PGE2-d4 chromatograph for Figure 4c; Figure S2: Internal standard PGE2-d4 chromatograph for Figure 4d; Figure S3: Full-length blots of manuscript Figure 2a; Figure S4: Full length blots of manuscript Figure 2d; Figure S5: Full length blots of manuscript Figure 3a; Figure S6: Full length blots of manuscript Figure 3c; Figure S7: Full length blots of manuscript Figure 4a; Figure S8: Full length blots of manuscript Figure 4b; Figure S9: Full length blots of manuscript Figure 5. Figure S10: Effects of PGE2 (a) and COX-2 inhibition (b) on intracellular NO levels in Sk-MEL-28 human melanoma cells. Experiments were independently repeated three times. Figure S11: Effects of NO stress (a) and nNOS inhibition (b) on PGE2 levels in SK-MEL-28 human melanoma cells as detected by LC-MS/MS. Experiments were independently repeated three times. Figure S12: In vivo tumor growth curve in volume mm^3^. Nude mice were injected with human melanoma A375 cells to induce tumor growth and treated with celecoxib 50 mg/kg/day p.o. for 23 days. Tumors were measured biweekly. Tumor growth is presented as tumor volume for each individual mouse. The average tumor volume is shown in red for control (*n* = 7) and blue for celecoxib (*n* = 7). The tumor growth curves of individual mice treated with celecoxib are represented in gray.
