# Supplementary material for: Crosstalk Between nNOS/NO and COX-2 Enhances Interferon-Gamma-Stimulated Melanoma Progression
Source: Cancers (Basel). 2025 Jan 31;17(3):477. doi: 10.3390/cancers17030477 (PMC11816268; doi:10.3390/cancers17030477)

Table S1: **Top 35 protein-probes and their coefficients from the *ASSIGN* prediction.**

Protein	Coefficients
Cox2-R-C	0.93193512
PD-L1-R-C	0.69464464
PAI-1-M-V	0.60697561
Rictor_pT1135-R-V	0.59576374
Aurora-B-R-V	0.59382328
Slfn11-G-C	0.54711178
FoxO3a-R-C	0.5373178
S6_pS240_S244-R-V	0.45321725
c-Myc-R-C	0.40465291
p38_pT180_Y182-R-V	0.38259304
HER2_pY1248-R-C	0.35120385
BMX-R-S	0.32819069
MAPK_pT202_Y204-R-V	0.32728354
PR-R-V	0.31778983
PMS2-R-V	0.29940596
Rb_pS807_S811-R-V	0.27022184
XBP-1-G-C	0.26044596
TIGAR-R-V	-0.9509685
ACC1-R-C	-0.7769894
UBAC1-R-V	-0.5666377
p38-MAPK-R-V	-0.5241002
ACC_pS79-R-V	-0.5116904
LDHA-R-C	-0.4885076
FoxM1-R-V	-0.4698613
Paxillin-R-C	-0.4482631
VASP-R-V	-0.4324834
p70-S6K1-R-V	-0.4104495
cdc25C-R-V	-0.4099164
RSK-R-C	-0.3514161
PI3K-p85-R-V	-0.3395832
HER3-R-V	-0.3320856
Cyclin-D3-M-V	-0.3229341
Pdcd4-R-C	-0.3165438
Gys-R-V	-0.2898496
WIPI2-R-C	-0.2679963

Table S2: Predicted IFN-γ treatment activity in three cell lines treated with vehicle, IFN-α, and IFN-γ. Derived from the RPPA data, the IFN-y protein signatures predicted IFN-y treatment activity in all 3 melanoma cell lines ranging from 0 and 1, where 0 indicates no activity and 1 means highest activity. Our signatures predicted an IFN-y activity score of 0 for all control treated cells. For IFN-α treated A375 cells, the mean activity was 0.008, indicating minimal activity. In contrast, the IFN-γ treated A375 samples exhibited a significantly higher mean activity of 0.935, reflecting strong treatment-induced protein expression changes. This was consistent with all 3 cell lines.

Cell Line	Treatment	Predicted IFN-γ activity in cell lines
A375	Control	0
	IFN-α	0.00841977
	IFN-γ	0.93508375
SK-MEL-28	Control	0
	IFN-α	0
	IFN-γ	0.9402131
WM115	Control	0
	IFN-α	5.28E-05
	IFN-γ	0.94580734

Table S3: IC_{50} values of HH044 and celecoxib from MTT assay in A375 human melanoma cells.

	Cytotoxicity (IC50 in A375 human melanoma) (µM)	Fold of HH044 IC_{50} alone
**HH044**	6.58	1
**HH044 + celecoxib 5µM**	5.38	0.82
**HH044 + celecoxib 15µM**	4.80	0.73
**Celecoxib**	16.7	NA

Figure S1: Internal standard PGE_2-d4 chromatograph for Figure 4b.

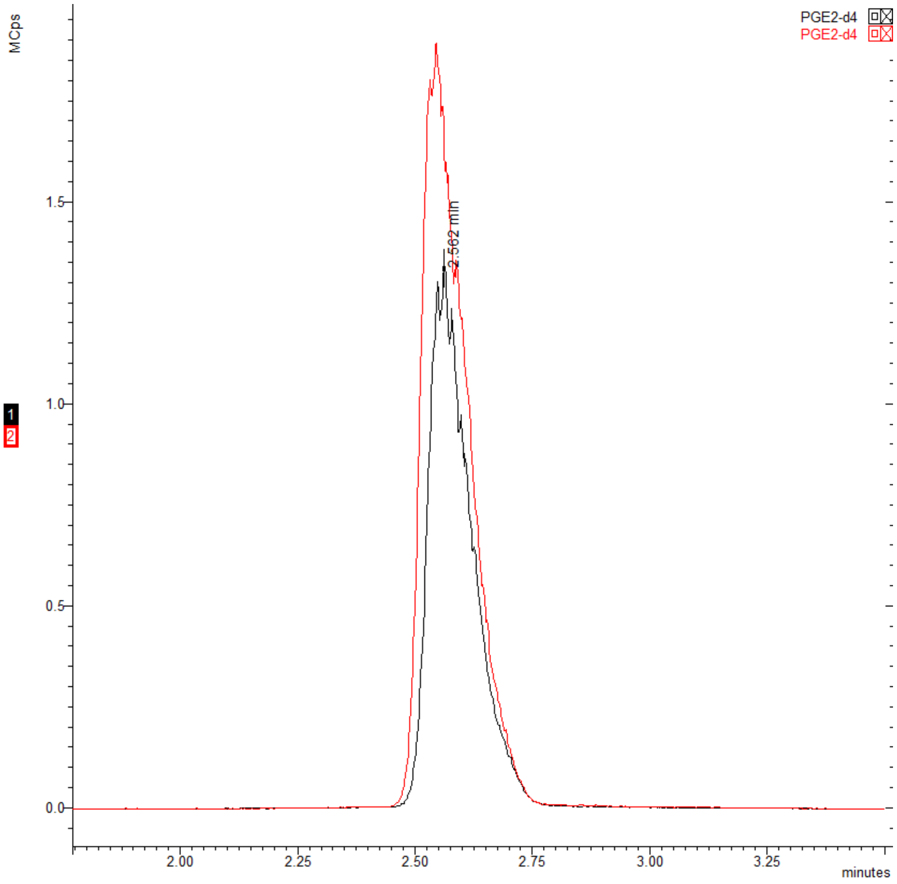

Figure S2: **Internal standard PGE_2-d4 chromatograph for Figure 4d.**

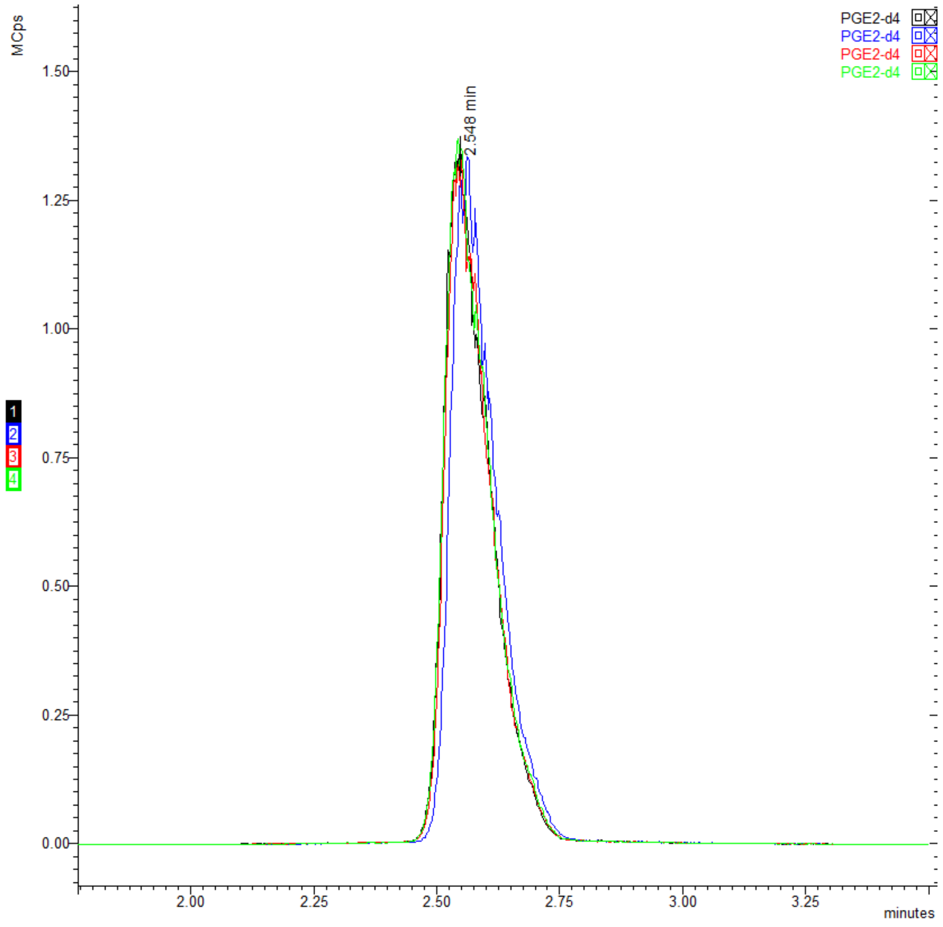

Figure S3: Full length blots of manuscript Figure 2a

	6h				24h				48h			
IFN-γ 100 U/mL	-	-	+	+	-	-	+	+	-	-	+	+
PGE_2 5μM	-	+	-	+	-	+	-	+	-	+	-	+

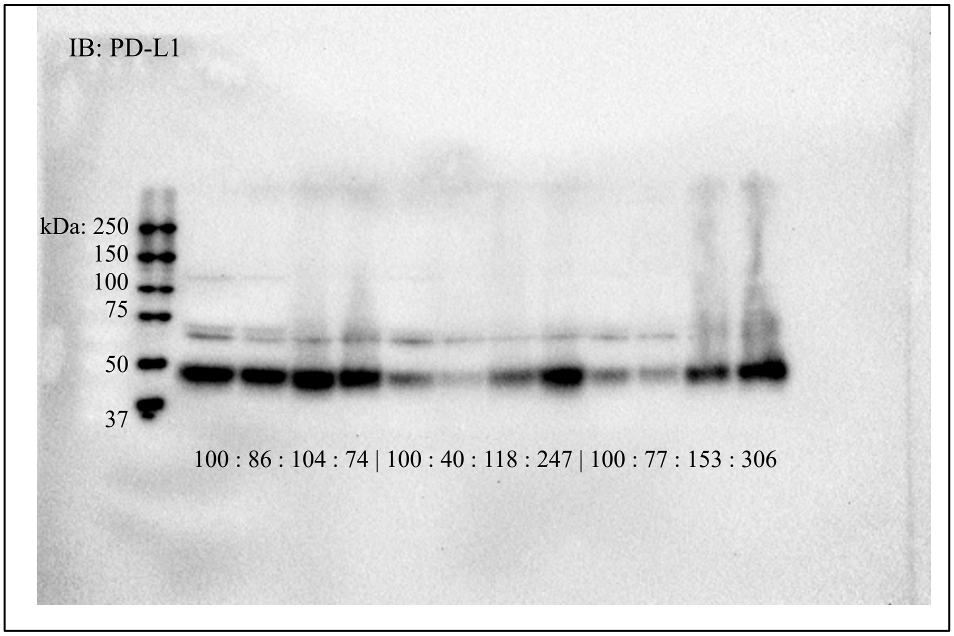

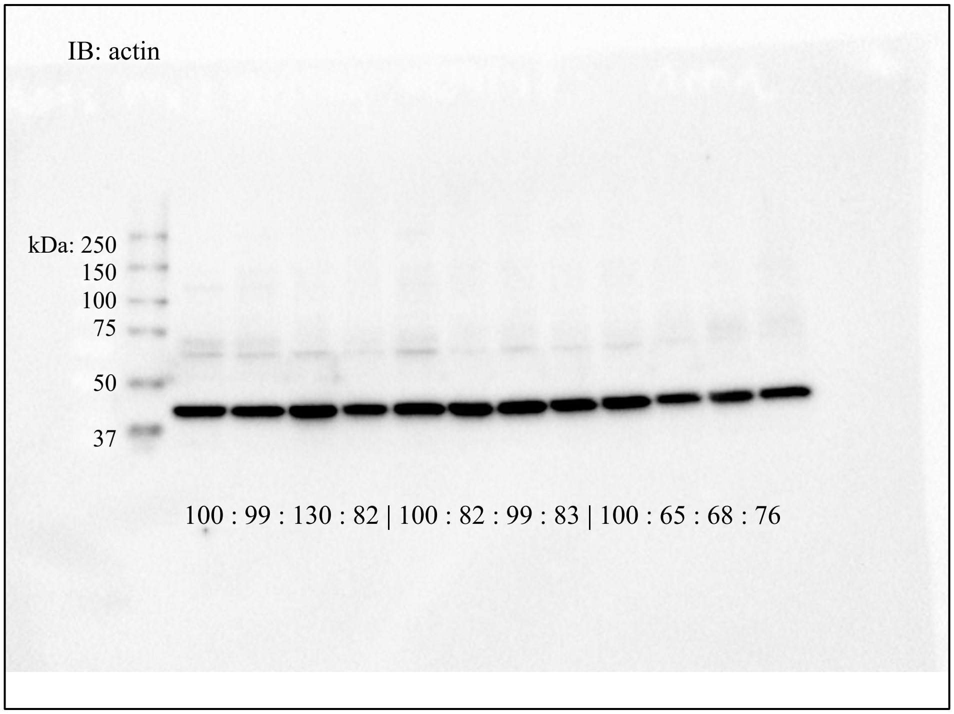

Figure S4: Full length blots of manuscript Figure 2d.

	24hr				48hr			
IFN-γ 250 U/mL	-	-	+	+	-	-	+	+
Celecoxib 50μM	-	+	-	+	-	+	-	+

IB: PD-L1

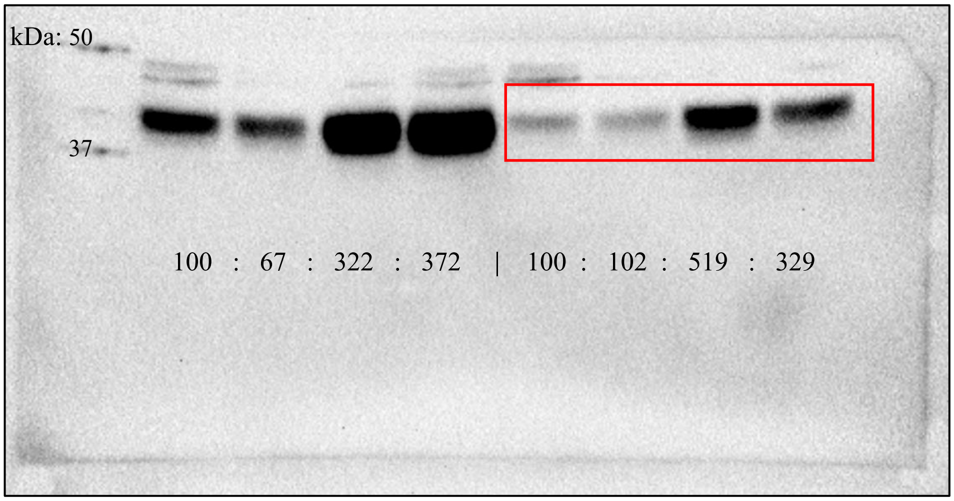

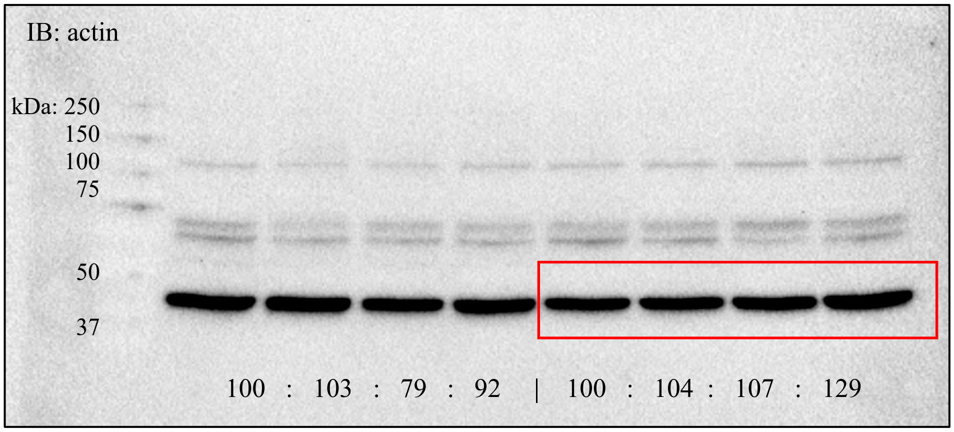

Figure S5: Full length blots of manuscript Figure 3a.

IFN-γ 250 U/mL	-	-	+	+	-	-	+	+
PGE_2	-	25μM	-	25μM	-	100μM	-	100μM

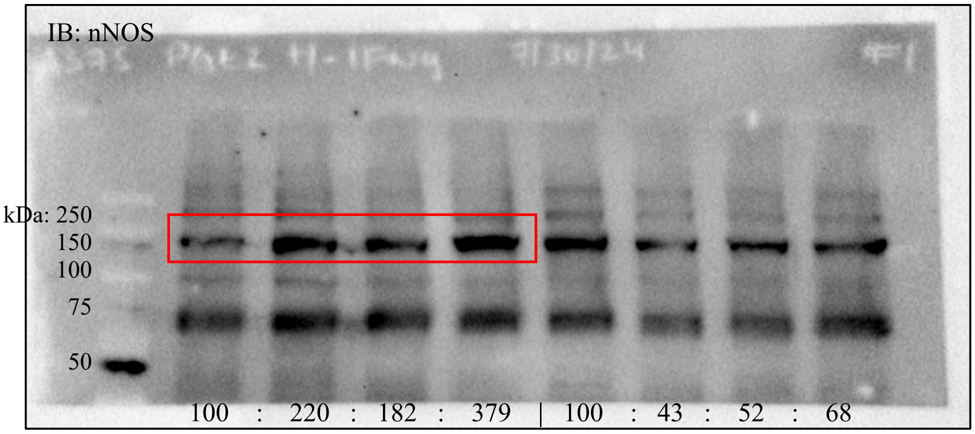

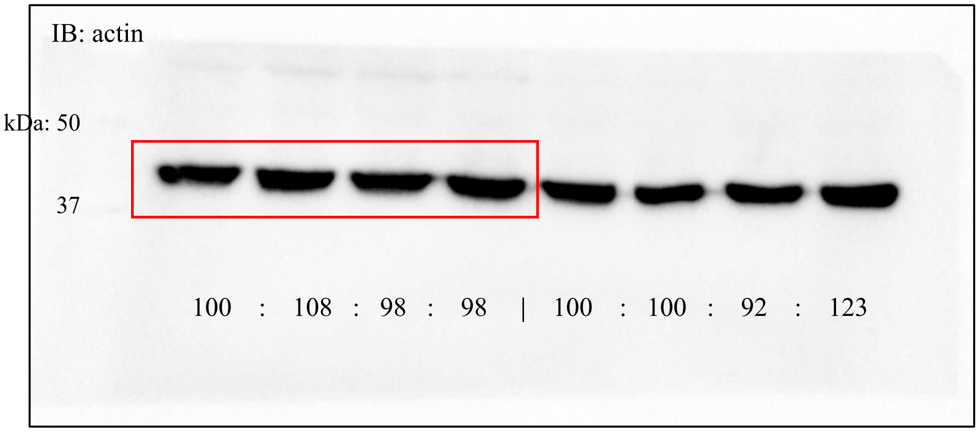

Figure S6: Full length blots of manuscript Figure 3c.

**IFN-γ 250 U/mL**	-	-	+	+
**Celecoxib 50μM**	-	+	-	+

IB: nNOS

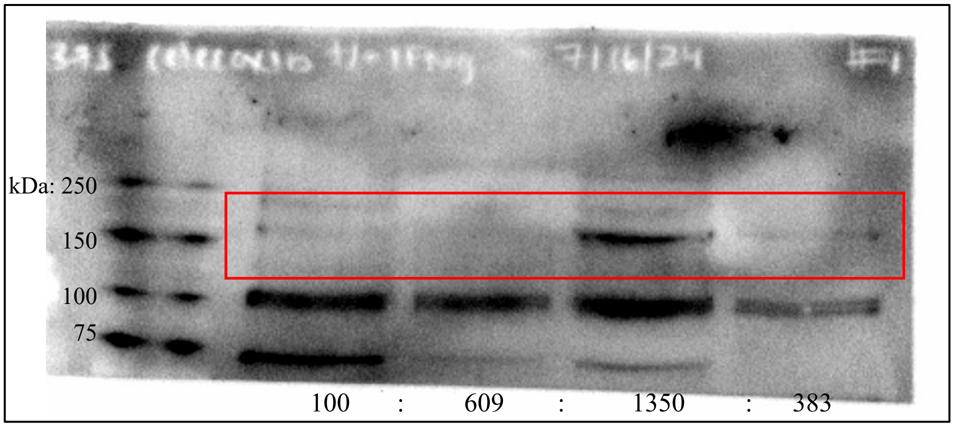

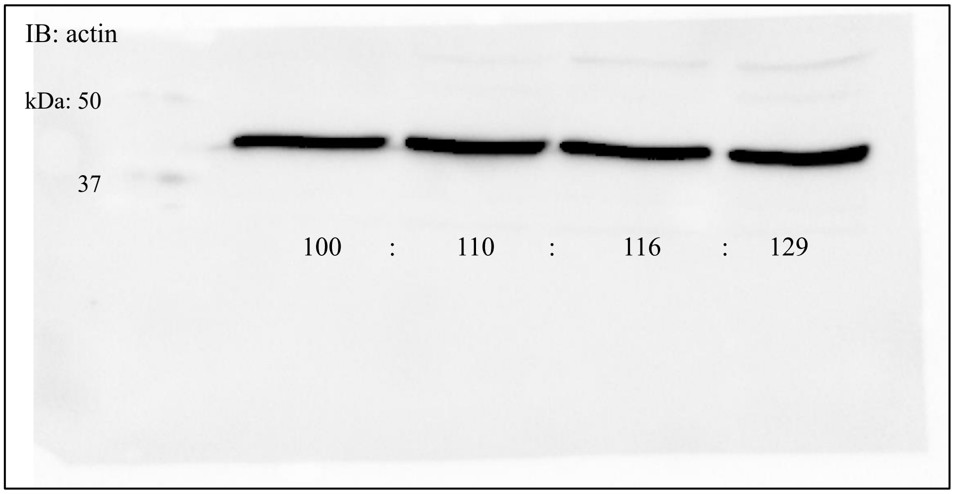

Figure S7: Full length blots of manuscript Figure 4a.

DetaNONOate 100μM	-	24 hours	48 hours

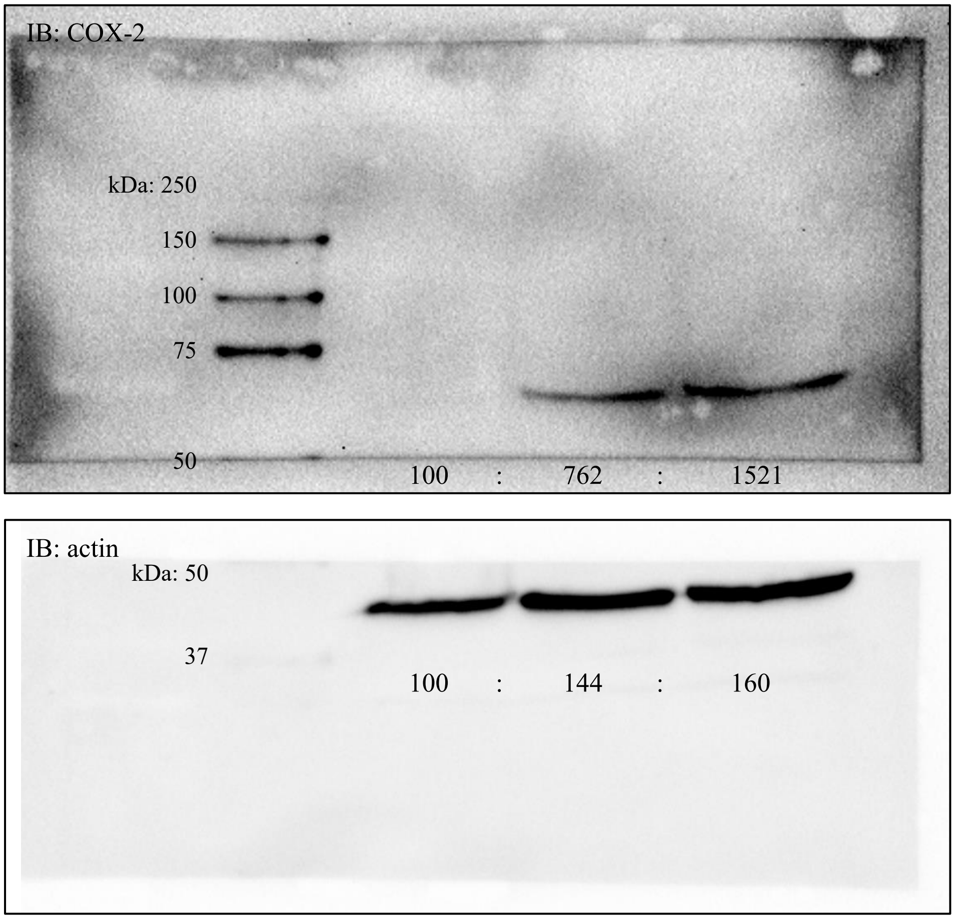

Figure S8: Full length blots of manuscript Figure 4b.

IFN-γ 250 U/mL	-	-	-	+	+	+
HH044	-	10μM	20μM	-	10μM	20μM

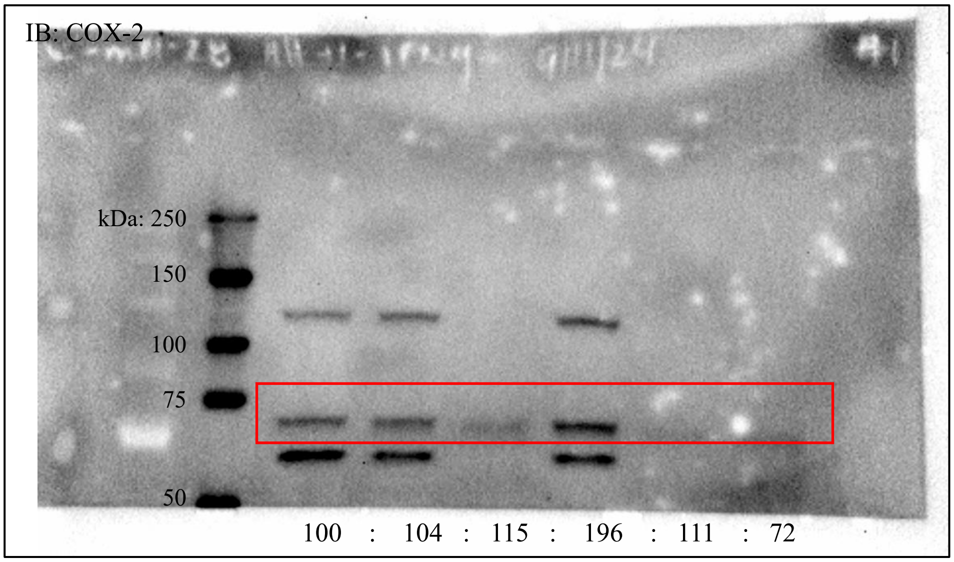

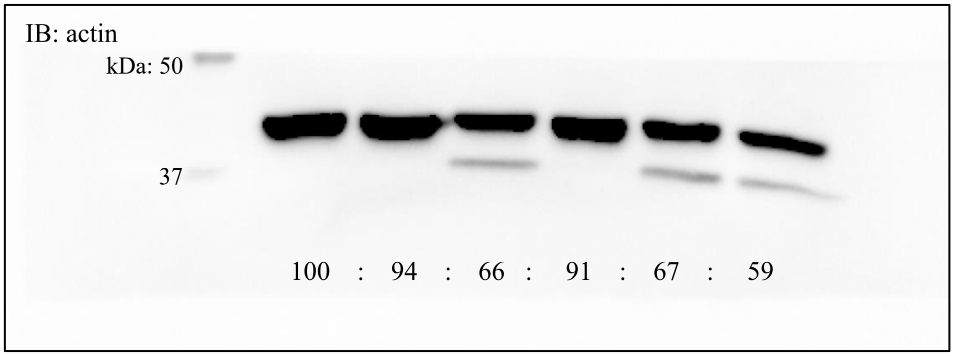

Figure S9: Full length blots of manuscript Figure 5.

IFN-γ 250 U/mL	-	-	+	+	-	-	+	+
Napabucasin 1μM	-	+	-	+	-	+	-	+

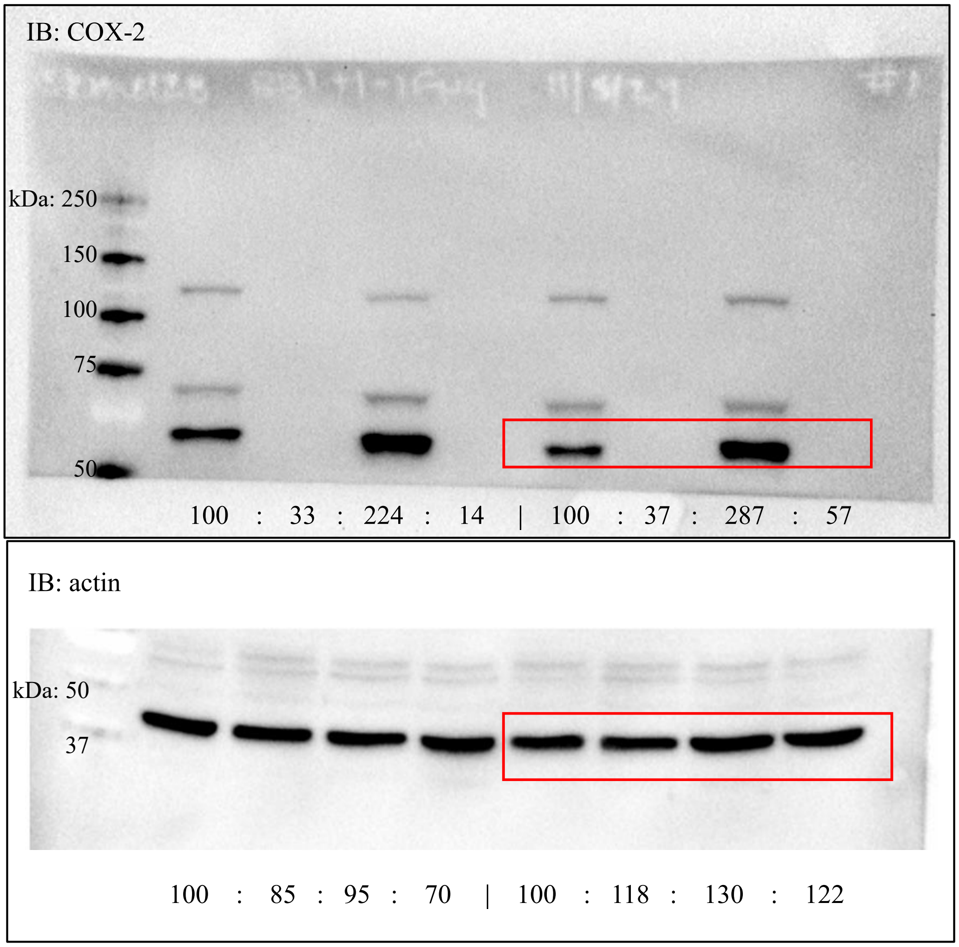

Figure S10: Figure S10: Effects of PGE_2 (a) and COX-2 inhibition (b) on intracellular NO levels in Sk-mel-28 human melanoma cells. Experiments were independently repeated three times.

a)

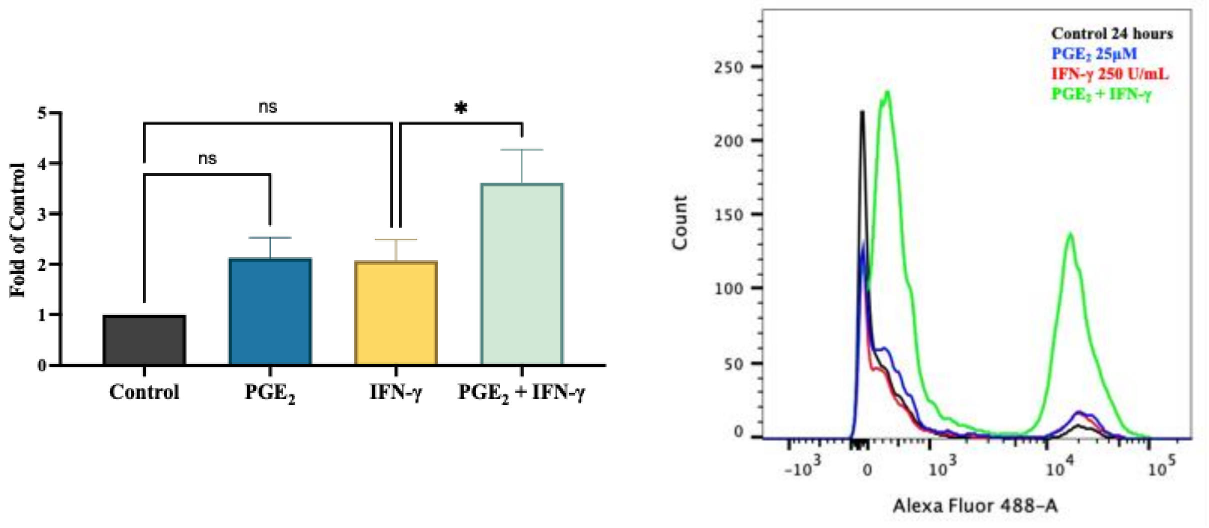

b)

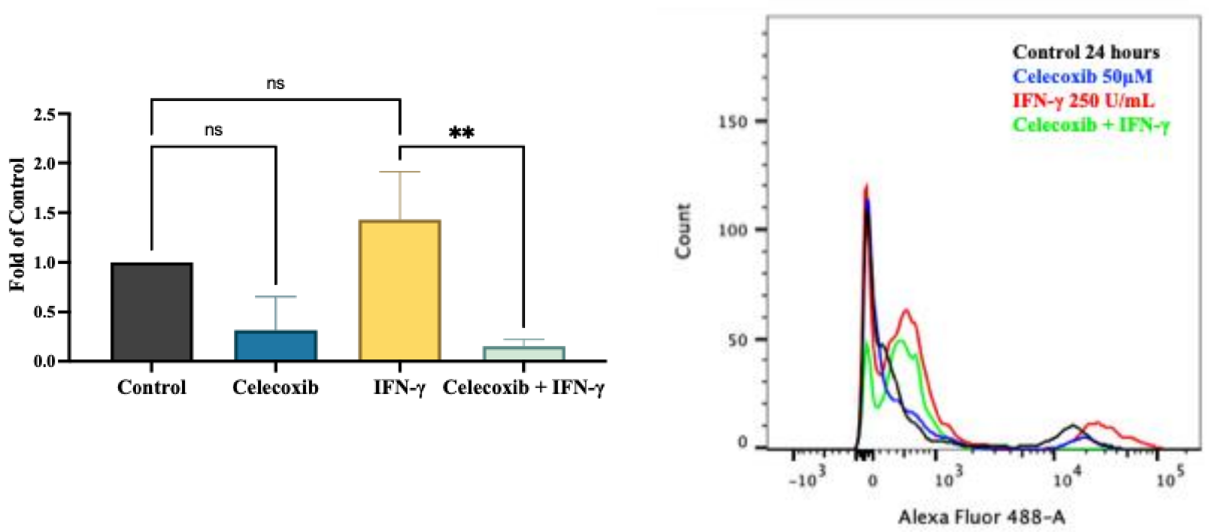

Figure S11: Effects of NO stress (a) and nNOS inhibition (b) on PGE_2 levels in SK-mel-28 human melanoma cells as detected by LC-MS/MS. Experiments were independently repeated three times.

a)

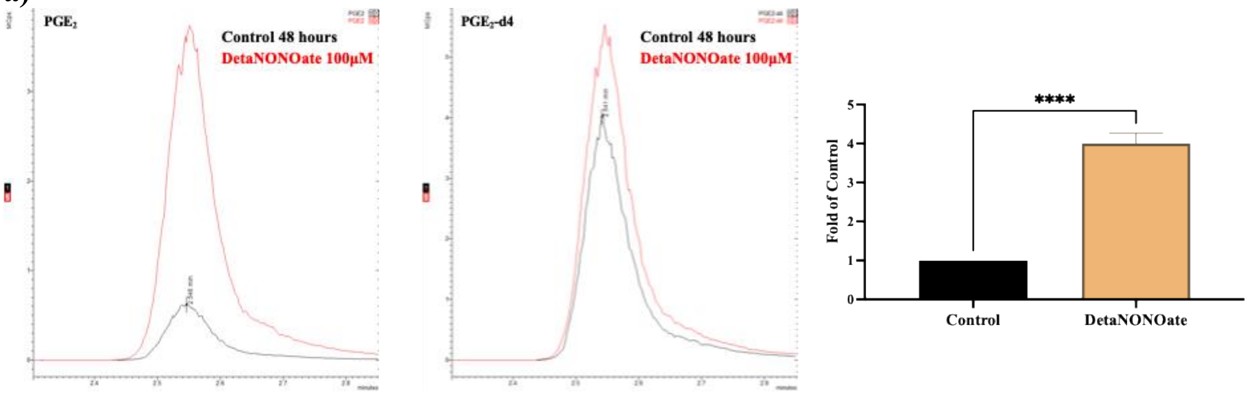

b)

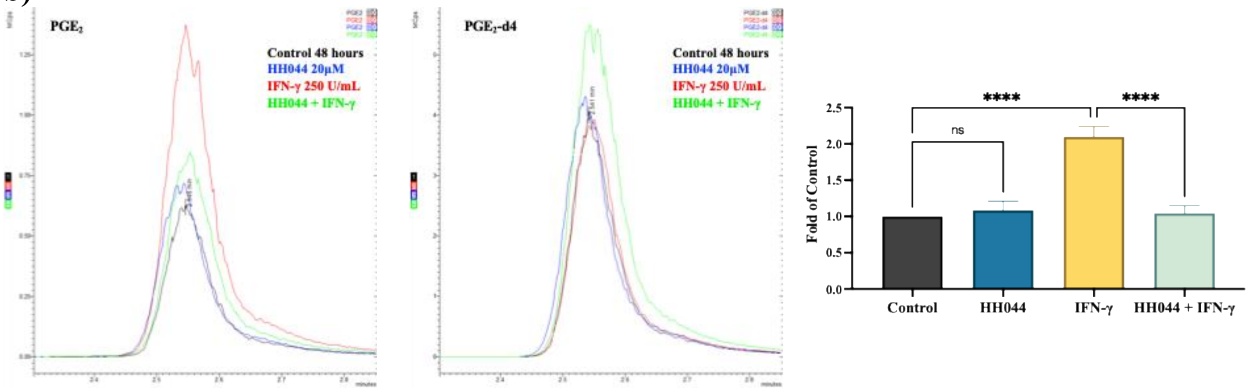

Figure S12: *In vivo* tumor growth curve in volume mm^3. Nude mice were injected with human melanoma A375 cells to induce tumor growth and treated with celecoxib 50 mg/kg/day *p.o.* for 23 days. Tumors were measured biweekly. Tumor growth is presented as tumor volume for each individual mouse. The average tumor volume is shown in red for control (n=7) and blue for celecoxib (n=7). The tumor growth curves of individual mice treated with celecoxib are represented in gray.

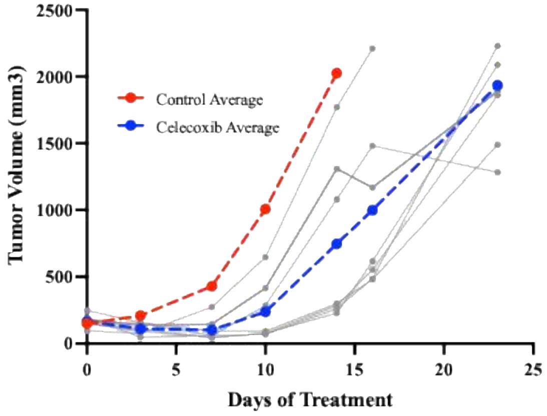

Supplement: Supplementary file 1 [file cancers-17-00477-s001.zip › cancers-3421005-supplementary.pdf]
